# Glucose and Fat Oxidation: Bomb Calorimeter Be Damned

**DOI:** 10.1100/2012/375041

**Published:** 2012-04-19

**Authors:** Christopher B. Scott

**Affiliations:** Department of Exercise, Health, and Sport Sciences, University of Southern Maine, 37 College Avenue, Gorham, ME 04038, USA

## Abstract

For both respiration and combustion, the energy loss difference between glucose and fat oxidation often is referenced to the efficiency of the fuel. Yet, the addition of anaerobic metabolism with ATP resynthesis to complete respiratory glucose oxidation further contributes to energy loss in the form of entropy changes that are not measured or quantified by calorimetry; combustion and respiratory fat/lactate oxidation lack this anaerobic component. Indeed, the presence or absence of an anaerobic energy expenditure component needs to be applied to the estimation of energy costs in regard to glucose, lactate, and fuel oxidation, especially when the measurement of oxygen uptake alone may incorrectly define energy expenditure.

## 1. Introduction

Antoine Lavoisier (1741–1794) provided a brilliant start. “La respiration est donc une combustion” he mused, well before thermodynamic distinctions were refined by acknowledging the type of fuel undergoing oxidation and almost 150 years prior to the founding of metabolic biochemistry: with glucose oxidation, 1 liter of O_2_ uptake *≈* 21.1 kJ; with fat oxidation, 1 liter of O_2_ uptake *≈* 19.6 kJ. From this description, the 1.5 kJ difference between substrates would appear to influence O_2_ consumption based on cellular energy demands [1]. As an example, 1000 kJ of energy would require the consumption of 47.4 liters of O_2_; with fat as a fuel, 51.0 liters are consumed. Yet, in some cases, substrate distinction is no longer considered because the measurement of O_2_ uptake in and of itself has been used to “quantify” energy costs. Direct measurements of heat loss—kJoules—are now rarely collected or reported. As this paper demonstrates, there is more than meets the eye in an O_2_-only estimate of energy exchange.

## 2. Glucose and Fat Oxidation: Combustion

The bomb calorimeter provides definitive proof of the energy differences among substrates. So, why is there a difference in heat loss per volume of O_2_ consumed between glucose and fat? For both oxidative respiration and combustion, the 1.5 kJ per liter of O_2_ difference between glucose and fat often is referenced to the efficiency of the fuel. To the contrary, William Thornton's law [2], published in 1917, reveals a rather remarkable similarity in heat loss among various gases undergoing combustion, with heat loss per O_2_ (and electron) equivalent more closely resembling a single constant in accordance with the energy equivalent of fat not glucose oxidation. In fact, fuel efficiency can differ among oxidized substrates. Variance in heat loss is indicative of the size of the molecules undergoing combustion. Larger molecules are likely to have more proportionate bond types, smaller molecules less, so that greater variability in heat (energy) loss is evident when smaller molecules like glucose are combusted and compared to larger ones like fat [3]. This also is true for respiratory oxidation. But there is more to a series of biochemical reactions as compared to the single oxidative step of combustion.

## 3. Glucose and Fat Oxidation: Respiration

**Figure 1:**
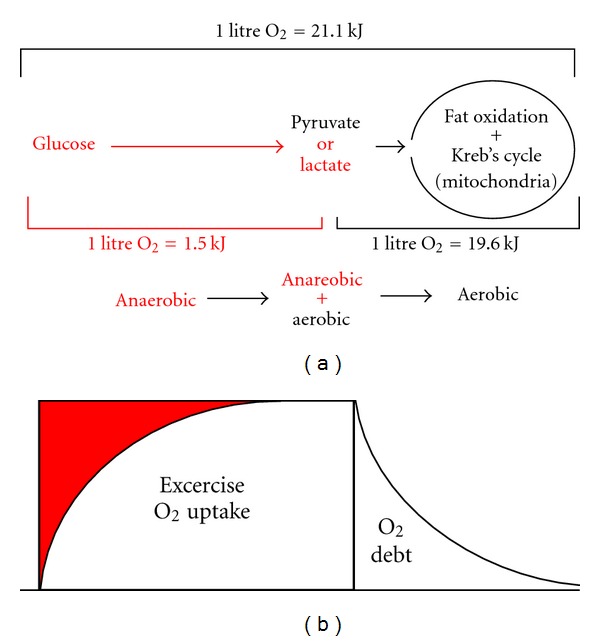
Anaerobic glycolytic metabolism is depicted in red, aerobic metabolism in black print, from left to right. At the top of the figure, both comprise a respiratory oxygen uptake to energy cost conversion of 1 liter of oxygen = 21.1 kJ when glucose is used as a fuel. Below, the anaerobic and aerobic components of a brief bout of exercise are shown, followed by an all-aerobic recovery (represented by the term “O_2_ debt”) where fat and lactate are consumed at 19.6 kJ per liter of oxygen. The 1.5 kJ per liter of O_2_ uptake difference is accounted for by entropy changes with pyruvate formation and by enthalpy changes when lactate is produced.

A quick flip through any biochemistry text offers a quite different explanation as to why respiratory glucose oxidation and energy loss would differ from that of fat: anaerobic glucose metabolism. Energy expenditure fueled by fat oxidation is entirely represented as mitochondrial O_2_ uptake. But before the aerobic oxidation of glucose derivatives within mitochondria, energy exchange known as *glycolysis* takes place that yields ATP yet consumes no oxygen (Figure 1(a)). And ultimately it is our quest to estimate ATP turnover that constitutes our need to quantify energy exchange. It appears, then, that in addition to the energy within the molecule itself, the metabolic processes of how complete respiratory glucose oxidation takes place also need deliberation [4]. In terms of anaerobic and aerobic energy loss, glycolytic ATP production is where respiration and bomb calorimetry (combustion) part ways.

In the 1970s, careful analyses of Gibbs energy (ΔG), enthalpy (ΔH), and entropy (ΔS) changes were completed as glucose was split into 2 molecules of pyruvate or lactate (and 2 net molecules of ATP were resynthesized) [5]. Gibbs energy changes were found to be identical regardless of what the “endproduct” of glycolysis was. With pyruvate as an end product, however, glycolytic Gibbs energy changes were largely accounted for by entropy; with lactate as the end product, Gibbs energy changes were detected by enthalpy (quantified as heat loss). This peculiarity between the two is important because entropy changes cannot be measured; they are instead calculated based on Gibbs energy (product/reactant ratios) and enthalpy measures. Thus, under aerobic conditions (i.e., pyruvate formation), glycolytic energy exchanges are identified by changes that are not typically considered part of an O_2_ uptake measurement. The 1.5 kJ difference per liter of O_2_ between respiratory glucose and fat oxidation acknowledges glycolytic energy exchange regardless of representation by heat or entropy. This theme can be readily applied to substrate utilization and O_2_ uptake during exercise and recovery.

## 4. Glucose, Lactate, and Fat Oxidation: Application

In addition to his Nobel-winning work disproving muscle as a heat engine (it was instead a chemomechanical converter), A.V. Hill suggested that the volume of the O_2_ debt incurred after the completion of exercise was affected by the amount of lactate produced and later removed [6]. The idea that lactate levels, in part, dictates the volume of O_2_ consumed caught on quickly. Note that O_2_ volumes are being quantified with this description, not energy expenditure based on substrate oxidation. For decades, lactate was thought causal to the O_2_ debt. Subsequent research however disproved the notion that lactate levels affect O_2_ consumption in recovery [7]. As a result, in 1984, exercise physiologists suggested a *qualitative* name change to replace the lactate-associated O_2_ debt hypothesis: excess postexercise oxygen consumption or EPOC [8].

Keep in mind that while glucose metabolism contains both an anaerobic and aerobic component, lactate oxidation is all aerobic. And the concept being demonstrated is not related to the thermic effect of feeding. Yet, separating glycolytic metabolism from mitochondrial O_2_ uptake introduces a methodology that recognizes a *quantitative* difference in glucose, fat, and lactate oxidation within cells. With glucose as a fuel, a muscles O_2_ uptake indeed reflects complete anaerobic plus aerobic glucose oxidation, at 1 liter O_2_ uptake = 21.1 kJ. Glycolysis with concomitant ATP production can subsequently be dismissed as part of respiratory O_2_ uptake measure in accordance with Thornton's combustion law, with lactate oxidation being equivalent to fat oxidation where there is no anaerobic component, at 1 liter O_2_ uptake = 19.6 kJ.

By representing glycolytic ATP resynthesis as a 1.5 kJ metabolic differential (per L O_2_ consumed) between glucose and fat/lactate oxidation, this energy cost component can be dismissed from an O_2_ debt measurement after the completion of exercise when fat and lactate oxidation are considered prevalent (Figure 1(b)). Does fuel utilization influence the O_2_ debt? First mentioned in the introduction, one interpretation states that fat oxidation *as compared to glucose oxidation* will indeed increase the volume of O_2_ consumed in recovery [9]. Why did not the previously mentioned lactate oxidation—O_2_ uptake studies promote this rationale? The answer appears to depend on whether the enthalpy and entropy energy exchange components of glycolysis and mitochondrial respiration are or are not considered part of an O_2_ uptake to energy expenditure estimation. In comparison with glucose, had lactate oxidation been expressed as kJ instead of by liters of O_2_, it would indeed have some influence on the volume of the O_2_ debt.

## 5. Synopsis

The concept of entropy, a thermodynamic parameter that is not measured via calorimetry, has been the focus of developmental biology for years [10]. Yet, the nutritional sciences have given little attention to this concept. One immediate application in the recognition of entropy is with the use of O_2_ uptake measurements to quantify the energy costs of living. In this regard, glucose oxidation has an anaerobic ATP resynthesis component that fat and lactate oxidations do not—this component is not measured with direct calorimetry when pyruvate is formed. Yet, it is there. And it needs to be accounted for if energy expenditure rather than O_2_ uptake is given consideration.
